# Inhibition of miR-153 ameliorates ischemia/reperfusion-induced cardiomyocytes apoptosis by regulating Nrf2/HO-1 signaling in rats

**DOI:** 10.1186/s12938-020-0759-6

**Published:** 2020-03-06

**Authors:** Wei Hou, Xianting Zhu, Juan Liu, Jiaguo Map

**Affiliations:** 1 Department of Emergency, Yidu Central Hospital of Wei Fang, No.4138, South Linglongshan Road, Weifang, 262500 Shandong China; 2 Department of Nursing, Yidu Central Hospital of Wei Fang, No.4138, South Linglongshan Road, Weifang, 262500 Shandong China; 3 Department of Pediatrics, Ward 1, Yidu Central Hospital of Wei Fang, No. 4138, South Linglongshan Road, Weifang, 262500 Shandong China; 4 Department of Cardiology, Qing Zhou Traditional Chinese Hospital, No. 2727, Haidai Middle Road, Weifang, 262500 Shandong China

**Keywords:** Ischemia–reperfusion, Rat, MiR-153, Nrf2, Adenoviral delivery

## Abstract

**Background:**

Previous in vitro studies demonstrated that suppression of microRNAs might protect cardiomyocytes and neurons against oxygen–glucose deprivation and reoxygenation (OGD/R)-induced cell apoptosis. However, whether the protective effect of miR-153-inhibition on cardiomyocytes can be observed in the animal model is unknown. We aimed to address this question using a rat model of ischemia–reperfusion (I/R).

**Methods:**

Rats were received the intramyocardial injection of saline or adenovirus-carrying target or control gene, and the rats were subjected to ischemia/reperfusion (I/R) treatment. The effects of miR-153 on I/R-induced inflammatory response and oxidative stress in the rat model were assessed using various assays.

**Results:**

We found that suppression of miR-153 decreased cleaved caspase-3 and Bcl-2-associated X (Bax) expression, and increased B cell lymphoma 2 (Bcl-2) expression. We further confirmed that Nuclear transcription factor erythroid 2-like 2 (Nrf2) is a functional target of miR-153, and Nrf2/Heme oxygenase-1 (HO-1) signaling was involved in miR-153-regulated I/R-induced cardiomyocytes apoptosis. Inhibition of miR-153 reduced I/R-induced inflammatory response and oxidative stress in rat myocardium.

**Conclusion:**

Suppression of miR-153 exerts a cardioprotective effect against I/R-induced injury through the regulation of Nrf2/HO-1 signaling, suggesting that targeting miR-153, Nrf2, or both may serve as promising therapeutic targets for the alleviation of I/R-induced injury.

## Background

Despite the fact that significant progress has been made in the treatment of cardiovascular diseases, they are still the most prevalent cause of death worldwide [1, 2]. Myocardial infarction is due to loss of blood flow resulting in local ischemia and subsequently causes cardiomyocytes injury. It is well accepted that re-establishment of blood flow together with reoxygenation in ischemic myocardial tissue is the most effective therapeutic approach for ischemic heart disease [3]. However, restoration of the blood flow and reoxygenation may induce innate inflammation, platelet activation, and excessive oxidative stress leading to even worse microstructural destruction termed myocardial I/R injury [4–10]. The mechanisms of myocardial I/R injury are not fully understood, and the practical strategy for preventing myocardial I/R injury is still limited.

miRNAs are small endogenous non-coding RNAs composed of 19–24 nucleotides that bind to imperfect sequence sites of target mRNA and recruit the RNA-induced silencing complex, causing either degradation or inhibition of protein translation [11]. Generally, one gene can be repressed by multiple miRNAs, and one miRNA may repress multiple target genes, so miRNAs are recognized as important regulators involved in various cellular processes, including differentiation, proliferation, necrosis, and apoptosis [12–14]. Recent studies have revealed that multiple miRNAs engage in the regulation of cardiovascular formation, development, and function [14]. For example, miR-21 exerts anti-apoptotic function via targeting Fas ligand or programmed cell death 4 (PDCD4) [15–17]. Overexpression of miR-21 in transgenic mice heart protected mice from severe heart failure through, at least in part, medicating Fas ligand expression [18, 19]. In line with this finding, Zhu et al. found that miR-499 enhances cardiomyocytes’ survival rate and protects the rat heart against I/R injury through targeting PDCD4 [20]. Recent evidence revealed that miR-153 was upregulated in response to I/R injury treatment [21, 22]. However, the precise role of miR-153 in the regulation of I/R-induced myocardial infarct remains elusive.

Nrf2/HO-1 signaling is a pivotal antioxidant signaling responsible for the maintenance of the balance of the redox state for the defense of intracellular oxidative stress [23, 24]. Excessive oxidative stress caused by I/R treatment activates the intrinsic apoptosis pathway leading to massive cell death. Thus, inhibition of high oxidative reactive species production can alleviate the I/R-induced damage. Importantly, activation of Nrf2/HO-1 signaling plays an important role in the inhibition of DNA damage and cell apoptosis by modulating oxidative stress [25, 26]. There is evidence showing that inhibition of miR-153 protects neurons against oxygen–glucose deprivation and reoxygenation (OGD-R)-induced injury by regulating Nrf2/HO-1 signaling [21]. The results from another group also suggested that suppression of miR-153 protected cardiomyocytes against OGD-R treatment by targeting Nrf2/HO-1 signaling [27]. Although the results from two groups are consistent and encouraging, these results were mainly performed on the OGD-R cellar model, and to validate these findings using an animal model is urgently needed.

In the current study, we applied the rat model of myocardial I/R injury to explore the functional role of miR-153 in I/R-induced myocardial infarct and the potential signaling pathway involved in this process. Our results, for the first time, demonstrated that suppression of miR-153 reduced I/R-induced inflammatory response and oxidative stress, enhanced cardiac function, and protected the heart from I/R-induced injury using a rat model. We further confirmed that Nrf2 is a functional target of miR-153, and Nrf2/HO-1 signaling was involved in miR-153-regulated I/R-induced cardiomyocytes apoptosis.

## Results

### Knockdown of miR-153 enhanced cardiac function

**Fig. 1:**
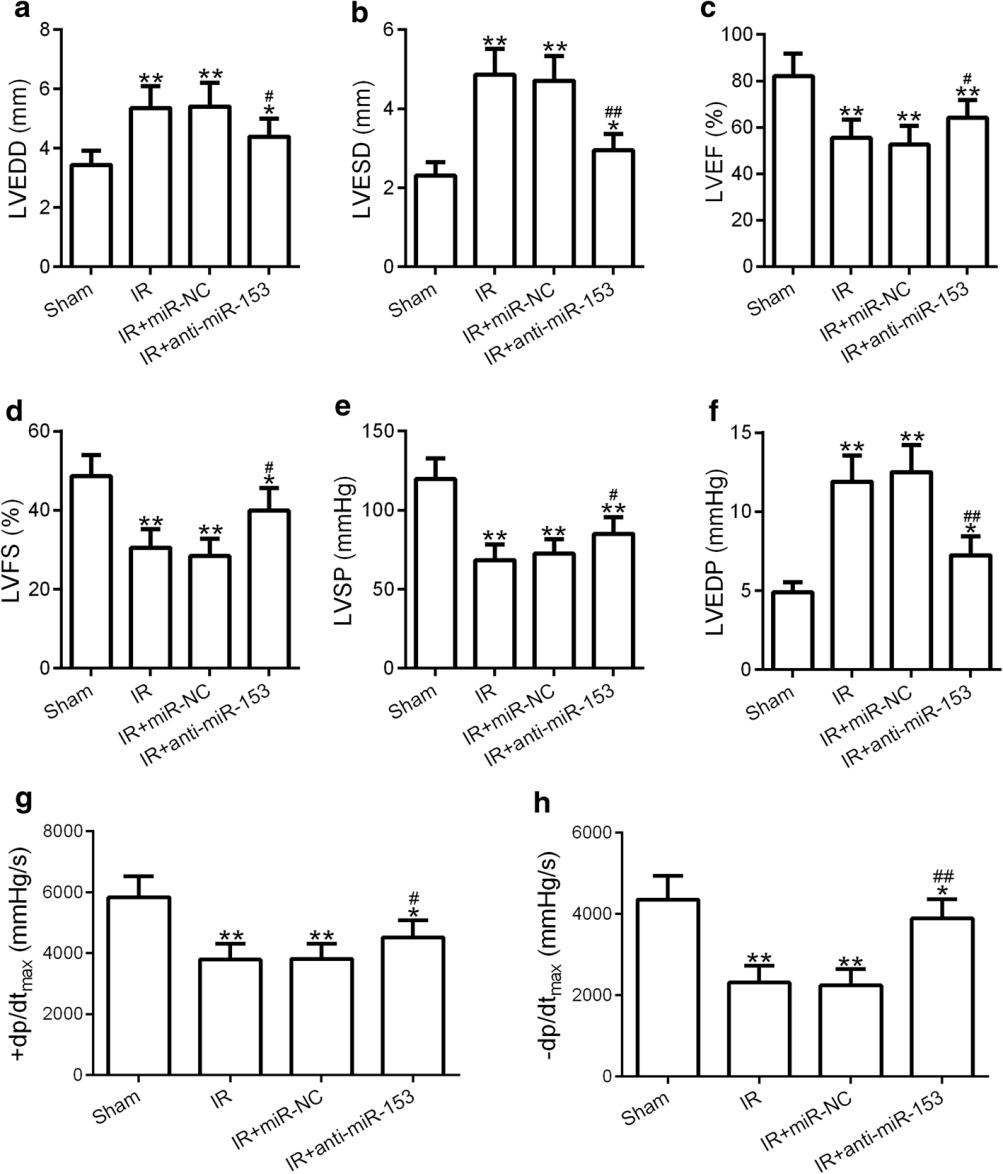
Inhibition of miR-153 ameliorated cardiac function in myocardial ischemia–reperfusion rat. Adult Sprague–Dawley rats were received the intramyocardial injection of saline (sham and I/R group), adenovirus-expressing miR-nonsense control (miR-NC), or anti-miR-153. Then, these rats were subjected to I/R treatment. LVEDD (**a**), LVESD (**b**), LVEF (**c**), LVFS (**d**), LVSP (**e**), and LVEDP (**f**) levels in the four groups were measured. **g**, **h** The influences of miR-153 on LV ± dp/dtmax were measured. *n* = 8 for each group. Experiments have repeated for three times independently. Data are presented as mean ± SD. *p < 0.05, **p < 0.01 compared to sham group. ^#^*p* < 0.05 and ^##^*p* < 0.01 compared to IR group. *LVEDD* left ventricular end-diastolic dimension, *LVESD* left ventricular end-systolic diameter, *LVEF* left ventricular ejection fraction, *LVFS* left ventricular fraction shortening, *LVSP* left ventricular systolic pressure, *LVEDP* left ventricular end-diastolic pressure

As illustrated in Fig. 1, the values of left ventricular end-diastolic dimension (LVEDD) and left ventricular end-systolic diameter (LVESD) were significantly enlarged in the I/R group compared with that in the sham group (Fig. 1a, b). Inhibition of miR-153, but not miR-NC, in I/R group (I/R + anti-miR-153) led to partially decrease the values of LVEDD and LVESD compared with that in the I/R group, but still statistically significantly higher than that in the sham groups. The values of left ventricular ejection fraction (LVEF) and left ventricular fraction shortening (LVFS) were markedly reduced in I/R group compared to sham group. Suppression of miR-153 in I/R group (I/R + anti-miR-153) led to partially restore the values of LVEF and LVES compared to the I/R group, but still lower than that in the sham groups (Fig. 1c, d). The values of left ventricular systolic pressure (LVSP) and the first derivative of the LV pressure (± dp/dt_max_) exhibited a similar tendency as the values of LVEF and LVFS (Fig. 1e, g, h), and the values of left ventricular end-diastolic pressure (LVEDP) showed a similar tendency as the values of LVEDD and LVESD (Fig. 1f). These results suggested that knockdown of miR-153 enhanced cardiac function and may protected myocardium from myocardial infarct.

### Suppression of miR-153 protected the heart against I/R-induced injury

**Fig. 2:**
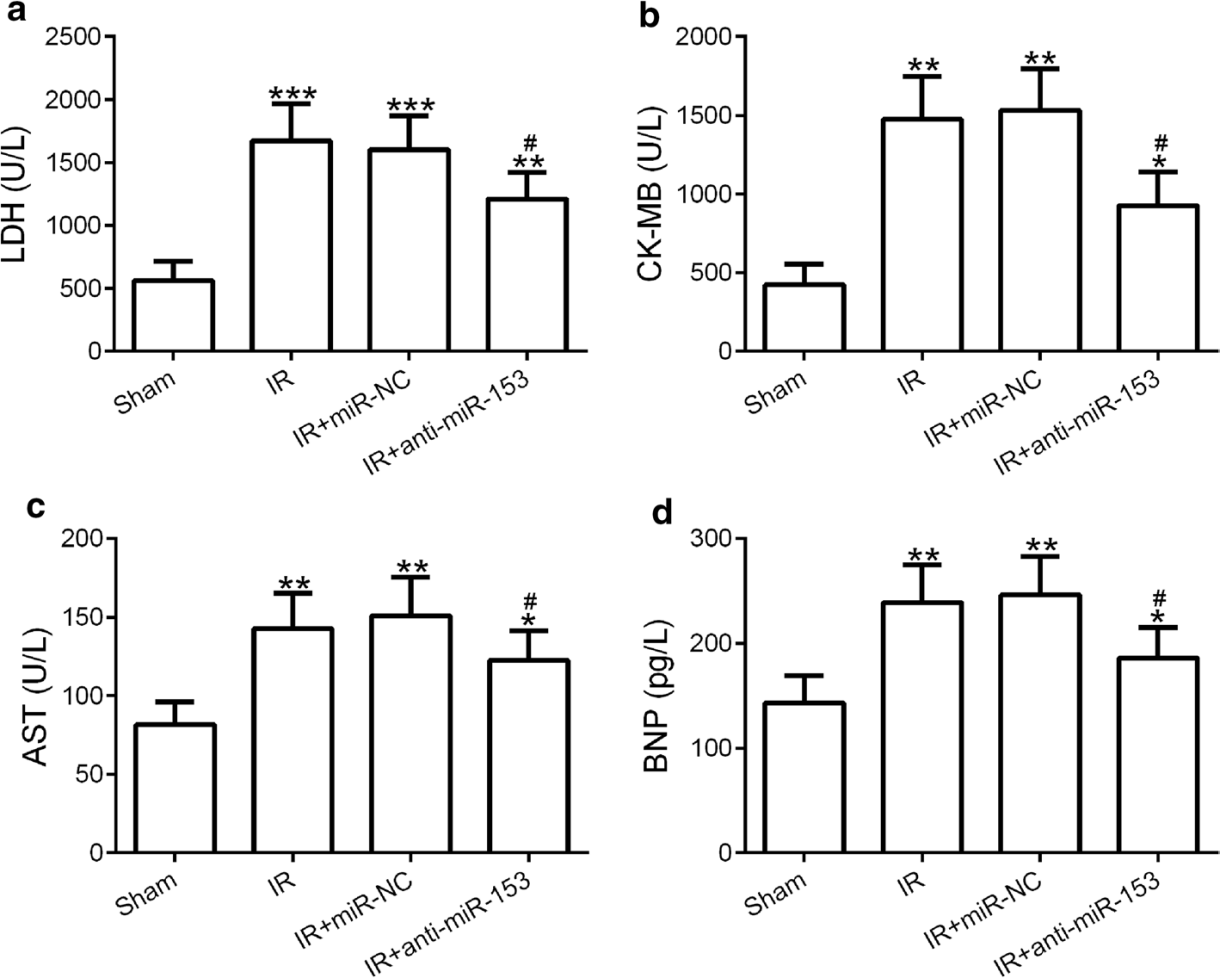
Knockdown of miR-153 decreased serum levels of LDH, CK-MB, AST, and BNP. Serum samples from rats as indicated in Fig. 1 were subjected to ELISA analysis of LDH (**a**), CK-MB (**b**), AST (**c**), and BNP (**d**). *n* = 8 for each group. Experiments have repeated for three times independently. Data are presented as mean ± SD. *p < 0.05, **p < 0.01, and ***p < 0.001 compared to sham group. ^#^*p* < 0.05 compared to IR group. *LDH* lactate dehydrogenase, *CK-MB* creatine kinase-MB, *AST* aspartate aminotransferase, *BNP* brain natriuretic peptide

To further confirm our hypothesis that anti-miR-153 may protect the heart against myocardial infarct, we collected serum samples from four groups of rat (sham, I/R, I/R + miR-NC, and I/R + anti-miR-153) and the expression levels of four proteins (BNP, Brain natriuretic peptide; CK-MB, creatine kinase-MB; AST, aspartate aminotransferase; LDH, lactate dehydrogenase) as the indicators for ischemia/reperfusion injury were measured [28, 29]. The expression levels of LDH were significantly enhanced in I/R treatment group and were partially reduced in miR-153-inhibited I/R + anti-miR-153 groups when compared with that in sham groups (Fig. 2a). In line with this observation, the other three proteins showed a very similar tendency as LDH (Fig. 2b–d). These results demonstrated that suppression of miR-153 indeed protected myocardium from I/R-induced myocardial infarct.

### Inhibition of miR-153 reduced I/R-induced inflammatory response and oxidative stress

**Fig. 3:**
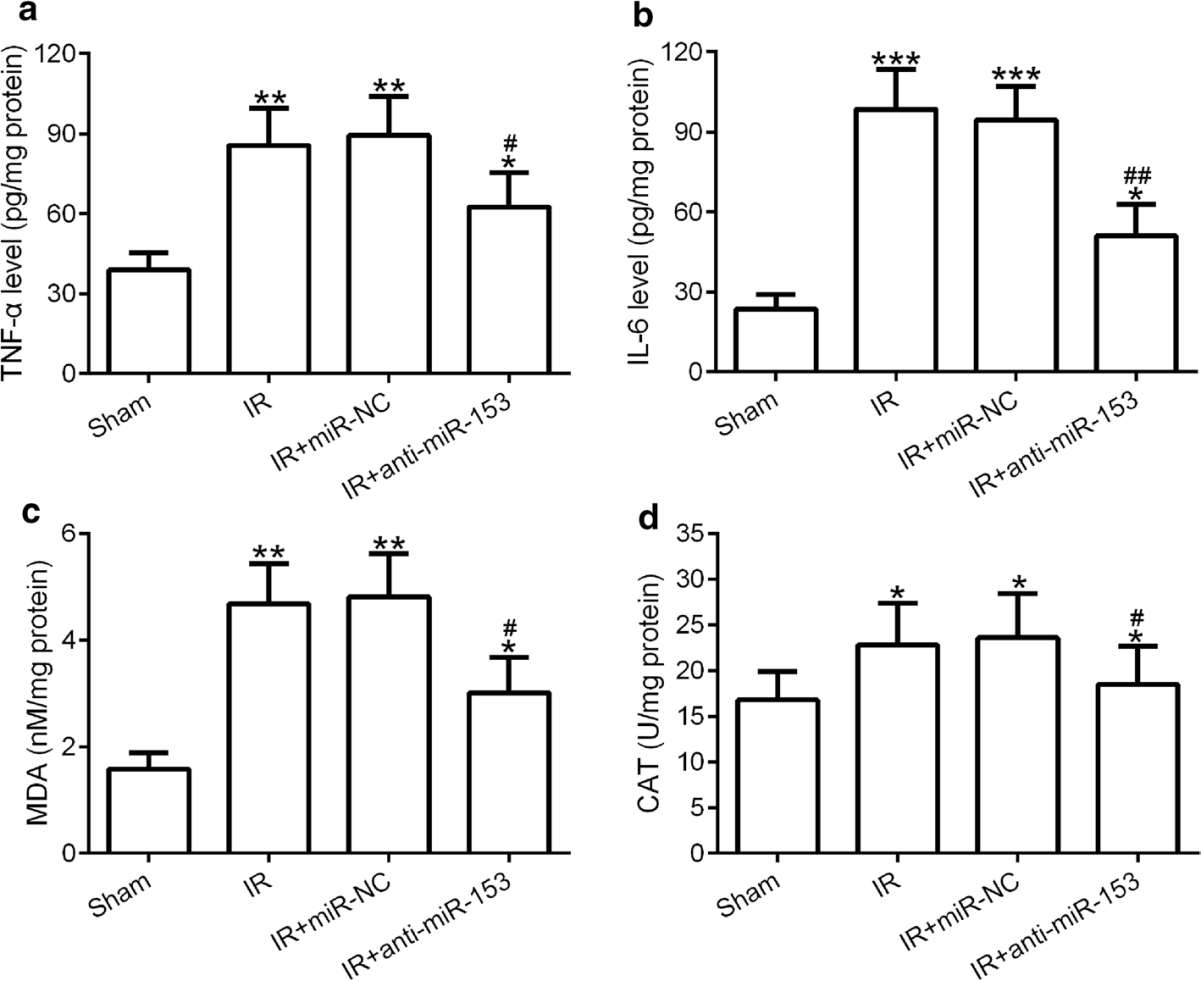
Inhibition of miR-153 decreased TNF-a, IL-6, MDA, and CAT expression. Myocardial inflammatory factors TNF-α (**a**) and IL-6 (**b**) were tested by ELISA. Myocardial oxidative stress factors MDA (**c**) and CAT (**d**) were tested by ELISA. *n* = 8 for each group. Experiments have repeated for three times independently. Data are presented as mean ± SD. *p < 0.05, **p < 0.01, and ***p < 0.001 compared to sham group. ^#^*p* < 0.05 and ^##^*p* < 0.01 compared to IR group

Excessive inflammatory response and oxidative stress are the crucial reason for causing myocardium injury after I/R procedure [7, 8]. To study the effect of anti-miR-153 on the inflammatory response and oxidative stress in myocardium induced by I/R procedure, we examined the expression levels of inflammatory factors (IL-6 and TNF-a) and oxidative stress markers (MDA and CAT) by ELISA. The results showed that I/R treatment dramatically increased the expression levels of TNF-a, IL-6, MDA, and CAT, and this promotion effect can be partially blocked by anti-miR-153 (Fig. 3a–d). These data suggested that inhibition of miR-153 reduced I/R-induced inflammatory response and oxidative stress in the myocardium.

### MiR-153 regulated I/R-induced cardiomyocytes apoptosis through Bax, Bcl-2, and caspase-3

**Fig. 4:**
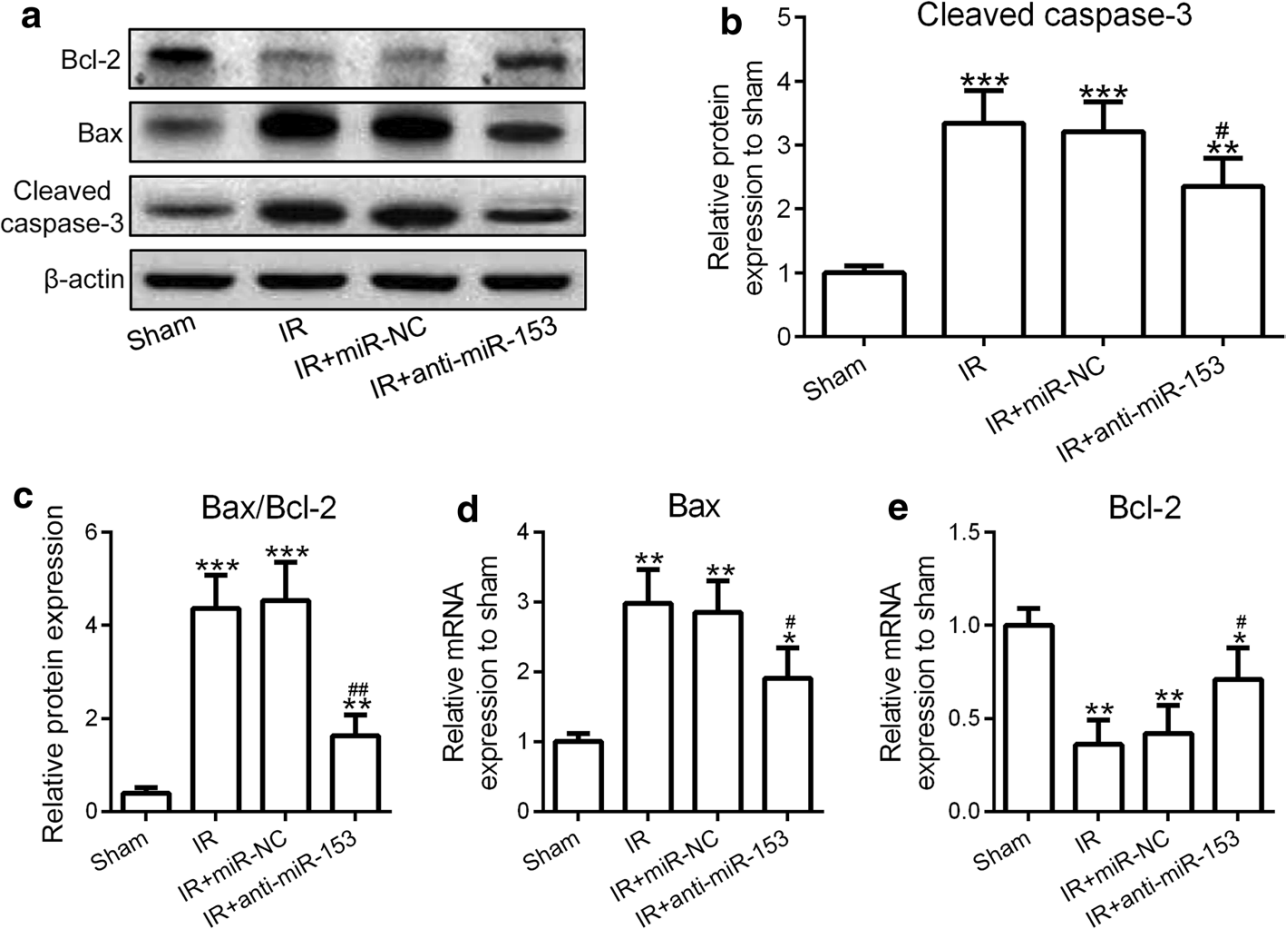
Suppression of miR-153 reduced cleaved caspase-3, Bax levels, and increased Bcl-2 levels. Heart protein samples from rats as indicated in Fig. 1 were subjected to western blotting analysis. **a** Western blotting results of the protein expressions of Bcl-2, Bax, and cleaved caspase-3. β-Actin was used as a loading control. Relative expressions of cleaved caspase-3 (**b**) and Bax/Bcl-2 (**c**) were normalized to the sham group. The intensity of the western blotting bands was quantified using image J. **d**, **e** The mRNA expressions of Bax and Bcl-2 were measured by RT-PCR. *n* = 8 for each group. Experiments have repeated for three times independently. Data are presented as mean ± SD. *p < 0.05, **p < 0.01, and ***p < 0.001 compared to sham group. ^#^*p* < 0.05 and ^##^*p* < 0.01 compared to IR group

To investigate the effect of anti-miR-153 on the I/R-induced cardiomyocytes apoptosis, we tested the expression levels of cleaved caspase-3 and Bax (two indicators for cell death) and Bcl-2 (an indicator for cell survival). We found that both mRNA and protein levels of Bax, as well as cleaved caspase-3 levels, were enhanced, while the Bcl-2 mRNA and protein levels were decreased following I/R treatment (Fig. 4a–e). Knockdown of miR-153 alleviated the effect of I/R on the upregulation of Bax and cleaved caspase-3 and downregulation of Bcl-2 (Fig. 4a–e). These results suggested that miR-153 regulated I/R-induced cardiomyocytes apoptosis through affecting Bax, Bcl-2, and caspase-3 expression.

### Nrf2 is a functional target of miR-153

**Fig. 5:**
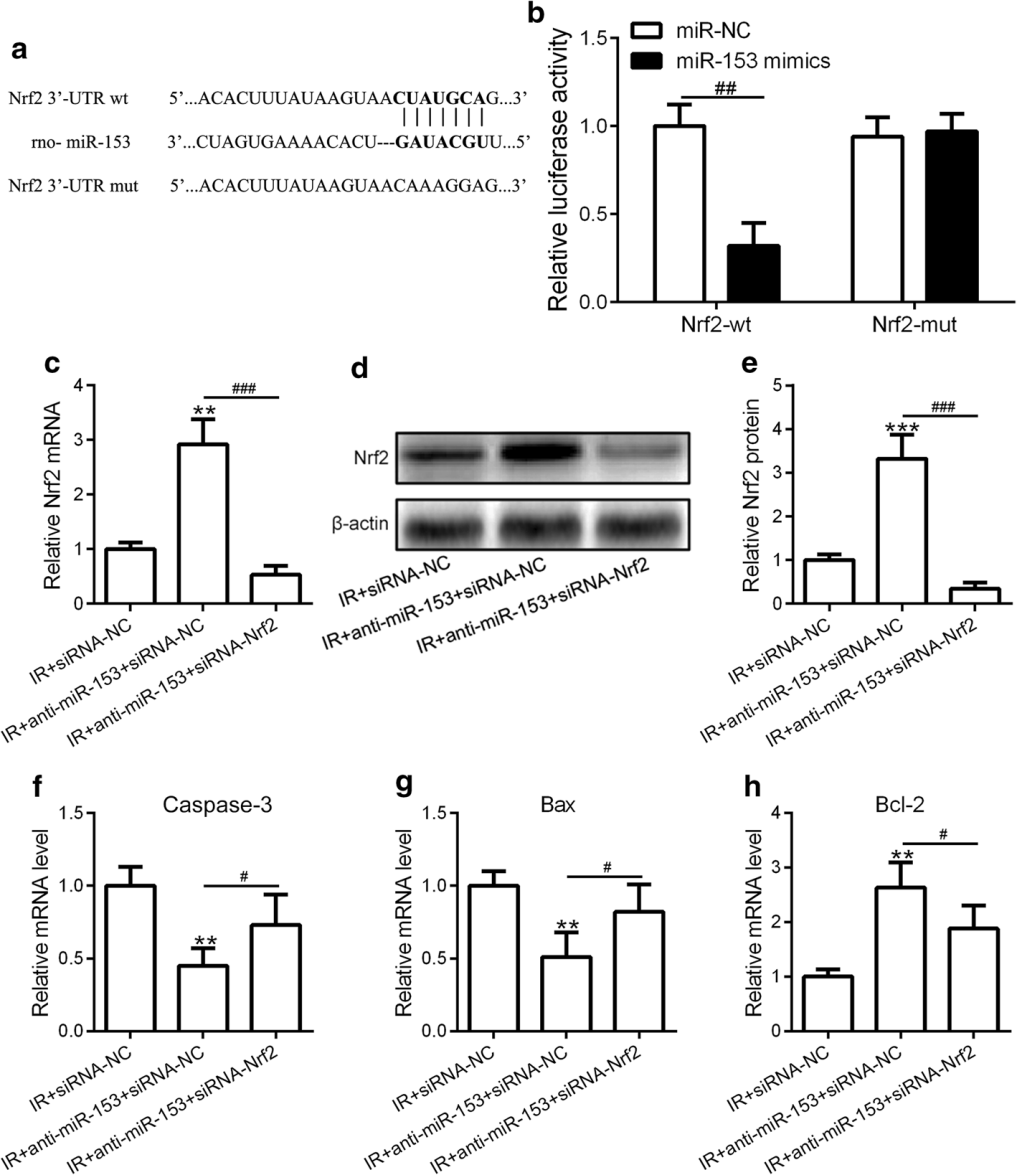
Nrf2 is a functional target of miR-153. **a** The putative binding of miR-153 with the wild-type or mutated 3′-UTR region of Nrf2 mRNA is shown. **b** HEK 293T cells were transfected with rat miR-153 mimics, miR-NC, pRT-TK Renilla, and Luc-Nrf2-wt or Luc-Nrf2-mut. After 48 h of incubation, relative luciferase activities were measured. Three independent experiments have been carried out. Sprague–Dawley rats were received the intramyocardial injection of saline, adenovirus-expressing siRNA-nonsense control (siRNA-NC), a combination of siRNA-NC and anti-miR-153 (anti-miR-153 + siRNA-NC), or a combination of siRNA-Nrf2 and anti-miR-153 (anti-miR-153 + siRNA-Nrf2). **c** The mRNA levels of Nrf2 in four groups of rats were measured by qRT-PCR. **d**, **e** The protein levels of Nrf2 in four groups of rats were measured by western blotting assay (**d**). The results were quantified by image J software (**e**). The mRNA levels of caspase-3 (**f**), Bax (**g**), and Bcl-2 (**h**) in four groups of rats were measured by qRT-PCR. *n* = 8 for each group. Experiments have repeated for three times independently. Data are presented as mean ± SD. **p < 0.01 and ***p < 0.001 compared to sham group. ^#^*p* < 0.05, ^##^*p* < 0.01, and ^###^*p* < 0.001

To further investigate the molecular mechanism of miR-153-regulated I/R-induced myocardial infarct, we applied bioinformatics analysis of the target gene of miR-153 using a well-established miRNA-target prediction tool (http://www.targetscan.org/vert_72/). Interestingly, we identified a small fraction of DNA sequence containing a potential binding site for miR-153 located in the 3′-UTR region of Nrf2 gene (Fig. 5a). To experimentally confirm this finding, we constructed two luciferase reporter plasmids containing the putative binding site of miR-153 or the mutated sequence. Overexpression of miR-153 significantly decreased the wild-type reporter activity, but not the mutant one, demonstrating that miR-153 can specifically target Nrf2 3′-UTR region by binding to its putative sequences (Fig. 5b). To investigate the biological function of Nrf2 in the miR-153-inhibition mediated I/R in vivo model, we knockdown of Nrf2 by introducing siRNA specifically targeting Nrf2 gene (Additional file 1: Figure S1). Indeed, inhibition of miR-153 led to upregulation of Nrf2 mRNA and protein levels, while siNrf2 transfection resulted in downregulation of Nrf2 mRNA and protein levels (Fig. 5c–e). We further showed that the inhibition effect of anti-miR-153 on caspase-3 and Bax was partially rescued by knockdown of Nrf2 and the promotion effect of anti-miR-153 on Bcl-2 was partially blocked in Nrf2-inhibited cells (Fig. 5f–h). These results strongly suggested that Nrf2 is a functional target of miR-153 in the mediation of I/R-induced cardiomyocytes apoptosis.

### Nrf2/HO-1 signaling was involved in miR-153-regulated I/R-induced cardiomyocytes apoptosis

**Fig. 6:**
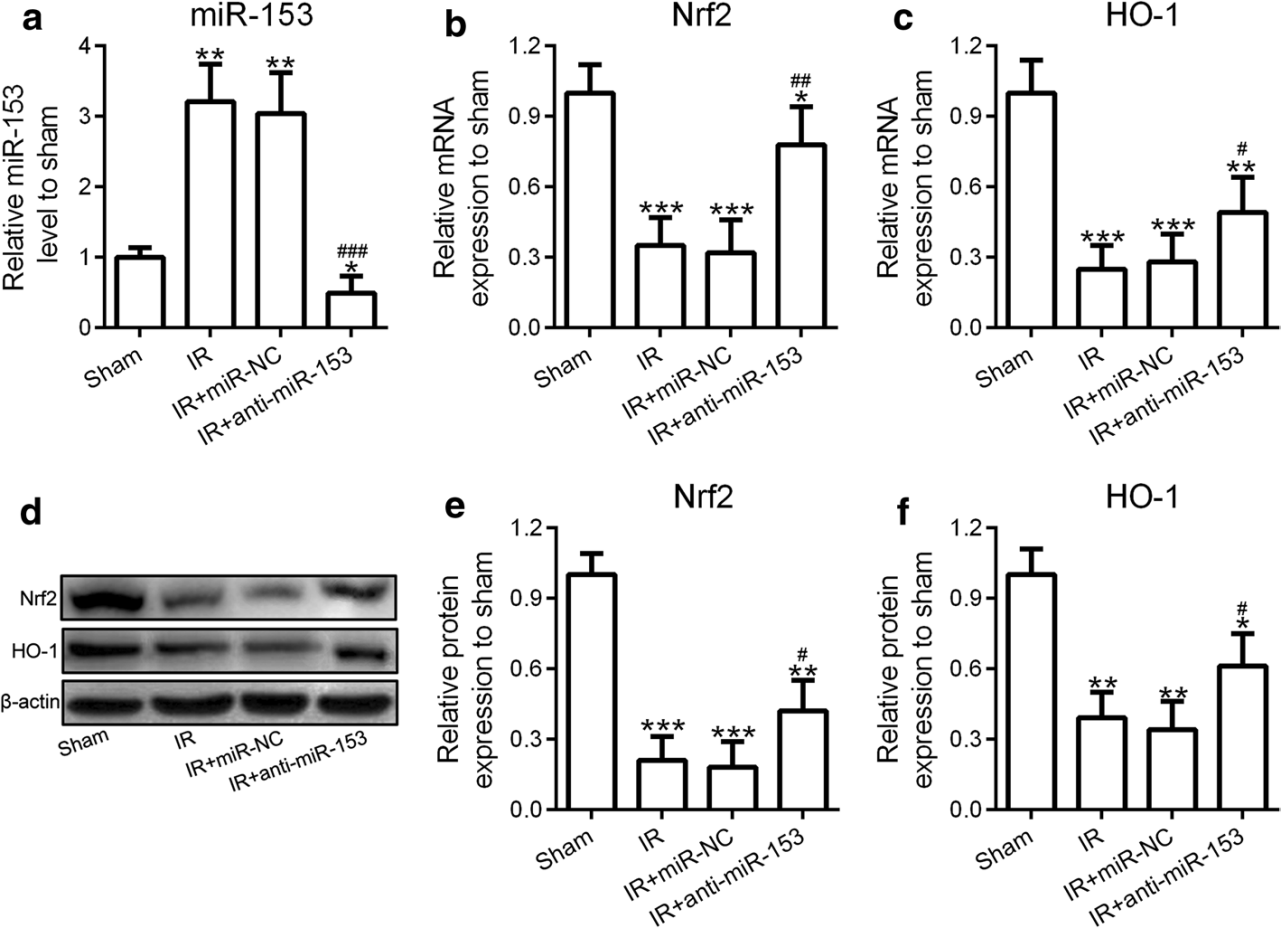
Nrf2/HO-1 signaling was involved in miR-153-mediated I/R-induced cardiomyocytes apoptosis. Rats were received intramyocardial injection of saline (sham and I/R group), adenovirus-expressing miR-nonsense control (miR-NC), or anti-miR-153. Then, these rats were subjected to I/R treatment. **a**–**c** The expression levels of miR-153 and mRNA levels of Nrf2, HO-1 were analyzed by qRT-PCR. The protein levels of Nrf2 in four groups of rats were measured by western blotting assay (**d**). The results were quantified by image J software (**e**, **f**). *n* = 8 for each group. Experiments have repeated for three times independently. Data are presented as mean ± SD. *p < 0.05, **p < 0.01, and ***p < 0.001 compared to sham group. ^#^*p* < 0.05, ^##^*p* < 0.01, and ^###^*p* < 0.001 compared to IR group

Nrf2/HO-1 signaling pathway plays a crucial role in the regulation of cell apoptosis [23, 27]. Since we showed that inhibition of miR-153 reduced I/R-induced cardiomyocytes apoptosis, we aimed to investigate the effect of miR-153 on Nrf2/HO-1 signaling. We designed three groups in this experiment, which were IR + siRNA-NC: a control group; siRNA-NC is a control for siRNA-Nrf2. We did not add miR-NC because we have shown that the results of IR and IR + miR-NC were similar. We found that I/R treatment induced high expression of miR-153 (Fig. 6a), which resulted in significantly decreased the mRNA and protein levels of Nrf2 and HO-1 (Fig. 6b–f). However, inhibition of miR-153 blocked I/R-induced miR-153 upregulation leading to partially restored mRNA and protein levels of Nrf2 and HO-1 (Fig. 6b–f). Collectively, these results suggested that miR-153 regulated I/R-induced cardiomyocytes apoptosis through directly targeting Nrf2/HO-1 signaling pathway.

## Discussion

Several therapeutic strategies to reduce or manage the myocardial I/R injury in patient have been proposed [30]. For example, for the hospital, it is developing a treatment protocol that minimizes the door-to-primary percutaneous coronary intervention time. Other modifications have also been proposed, including improving earlier reperfusion, development of better percutaneous coronary intervention technology, modifications of the reperfusate composition, and administration of free radical scavengers, antiplatelet and antithrombotic agents [31, 32]. Despite the progresses in the management of myocardial I/R injury, the underlying mechanisms of I/R-induced myocardial damage is not fully reveled. In this study, we investigated the role of miR-153 in I/R-induced myocardial damage using a rat model of ischemia–reperfusion.

Due to the complex nature of miRNAs, miR-153 can function as both tumor promotor and tumor suppressor in various human cancers. For example, as a tumor suppressor, miR-153 inhibits the migration and tuber formation of breast cancer cells by blocking angiopoietin 1 [33]. However, as an oncogene, miR-153 promotes the cell proliferation of prostate cancer via suppression of phosphatase and tensin homolog (PTEN), and upregulation of miR-153 is associated with a poor prognosis of prostate cancer patients [34].

Although the pathophysiological mechanisms underlying I/R injury are not fully understood, the excessive oxidative stress is a key initiator of I/R injury. Reactive oxygen species overload can overwhelm the intracellular antioxidant system, leading to apoptosis and tissue damage [35, 36]. Several publications revealed that miR-153 is involved in the regulation of oxidative stress response through modulation of Nrf2 in bovine granulosa cells [37], breast cancer cells [38], and neurons. However, the roles of miR-153 in I/R-induced high oxidative stress-mediated myocardial injury in the animal model remain unknown.

Surgical coronary ligation is a useful experimental technique to induce models of myocardial infarction in various animal types, including mouse, rat, dog, and pig [39–41]. First, because I/R treatment enhanced miR-153 expression in the heart, we knock-down of miR-153 via overexpressing anti-miR-153 in the heart of rat via adenovirus infection. Gene therapy is a powerful modality in the treatment of human diseases. The adenoviral vector is a desirable tool for a rapid, transient, and robust expression of target gene in the desired area or organ [42, 43]. Intramyocardial injection of adenovirus resulted in a lower inflammatory response and higher transgene expression compared to systemic tail vein injection [44, 45]. Indeed, we found that adenovirus injection led to local and rapid downregulation of I/R-induced miR-153 in the heart area. Overexpression of anti-miR-153 blockaded the I/R-induced miR-153 upregulation resulting in enhanced cardiac function and protected myocardium against I/R-induced injury. Second, we showed that intramyocardial injection of adenovirus overexpressing anti-miR-153 did not stimulate severe immune responses in the rat, suggesting that the intramyocardial injection of adenovirus is a useful surgical procedure for the rat. We further found that inhibition of miR-153 reduced I/R-induced inflammatory response and oxidative stress in myocardium. Third, we verified that Nrf2 is a functional target of miR-153. Nrf2 is a transcription factor that plays a major role in the dynamic regulation of a network of antioxidant and cytoprotective genes. Nrf2 is indicated in playing a role in different aspects of cardiovascular disease [46–48]. Applied the same surgical strategy, we delivered siRNA targeting Nrf2 into the heart of rat using adenovirus injection. We found that the suppression of miR-153 abrogated I/R-induced cell death signaling protein (i.e., caspase-3 and Bax) upregulation and cell survival protein (i.e. Bcl-2) downregulation, leading to protected rat heart from I/R-induced injury. However, knockdown of Nrf2 in anti-miR-153-expressing groups resulted in restored caspase-3 and Bax upregulation and Bcl-2 downregulation upon I/R treatment and subsequently impaired the protective effect of anti-miR-153 on the heart.

Although our current finding is exciting, there are a few questions that need to be addressed in future studies. For example, besides miR-153, whether other miRNAs are also involved in the regulation of I/R-induced injury? Whether an additional target of miR-153 plays a role in miR-153-mediated oxidative stress levels? To make our finding more applicable, a larger number of animals with diverse in age are urgently needed to further verify our results. In addition, a reasonable modification on animal experimental procedures, such as exposure time, or operation type, will also be beneficial for reducing the pain or damage to animals and improving the success rate.

## Conclusion

Based on our results from a rat model of ischemia–reperfusion, we confirmed the ideal derived from in vitro experiments that inhibition of miR-153 protects the heart against I/R-induced injury through regulation of Nrf2/HO-1 signaling. We also demonstrated that intramyocardial injection of adenovirus is a useful surgical procedure for the rat. Overall, our data from the rat model of I/R clearly demonstrated that suppression of miR-153 indeed protected myocardium against I/R-induced injury.

## Materials and methods

### Animals

Adult male Sprague–Dawley rats weighing 250–300 g (specific pathogen-free grade) were purchased from Nanjing Model Animal Institute. All work were performed under animal protocols approved by the Institutional Animal Care and Use Committee of Qing Zhou Traditional Chinese Hospital and complied with the Guide for the Care and Use of Laboratory Animals. To investigate the role of anti-miR-153 on cardiac function in vivo, we overexpressed anti-miR-153 in the heart of rat via adenovirus infection (Additional file 1: Figure S1). We had four groups in this experiment, which were sham: negative control; IR: positive control; IR + miR-NC: control for anti-miR-153; IR + anti-miR-153: this is the treatment group. After the ischemia/reperfusion experiment, the mice were examined by color Doppler echocardiography.

### Quantitative real-time PCR analysis

Total RNA was isolated with TRIzol assay (Invitrogen, Pleasanton, CA) following the manufacturer’s protocols. Isolated RNA was reversed transcribed using ABI TaqMan RT kit (Applied Biosystems, Waltham, MA). The pre-designed primer to detect rat miR-153 (Assay ID: 001191), rat Nrf2 (Assay ID: Rn00477784_m1), Bcl-2 (Assay ID: Rn00597992_m1), Bax (Rn01480161_g1), and caspase-3 (Rn00563902_m1) was purchased from Thermo Fisher Scientific. U6 and Glyceraldehyde 3-phosphate dehydrogenase (GAPDH) expression were used to normalize gene expression for miRNA and genes mRNA, respectively. Quantitative real-time PCR (qRT-PCR) was performed using an SYBR green/fluorescein or Taqman qPCR Master Mix on an ABI Prism 7500 system (Applied Biosystems).

### Western blotting assay

Cells were washed and lysed in RIPA lysis buffer with protease inhibitors (Thermo Fisher Scientific, Waltham, MA). The total proteins were separated by 8% or 10% gradients sodium dodecyl sulfate–polyacrylamide gel electrophoresis. Proteins were transferred to a polyvinylidene difluoride membrane and blocked with 5% non-fat milk. Then, the membrane was incubated overnight with primary antibodies. Protein bands were detected by incubation with horseradish peroxidase-conjugated antibodies and visualized with an enhanced chemiluminescence reagent. Antibodies against rat Nrf2, HO-1, Bcl-2, Bax, Cleaved caspase-3, and b-actin were purchased from Cell Signaling Technology (Danvers, MA). The intensity of the western blotting bands was quantified using image J.

### Adenoviral vector construction and intramyocardial injection

The adenoviral vectors were purchased from Genechem (Shanghai, China). The anti-miR-153, miR-nonsense control (NC), siNrf2, or si-nonsense control (NC) oligonucleotides were cloned into the AdMax adenovirus system (Microbix Biosystems, Mississauga, Canada). Recombinant adenoviruses were produced in HEK293 cells (American Type Culture Collection, VA, USA). The cell-culture medium containing adenovirus was then purified using an Adeno-XTM Virus Purification Kit (BD Biosciences; Clontech, CA), titrated to achieve 2.0 × 10^10^ Plaque forming units (PFU)/mL, and stored at − 80 °C.

Adult Sprague–Dawley rats (250–300 g) were anesthetized with sodium pentobarbital at a dose of 100 mg/kg. The heart was exposed upon opening the left pleural cavity by cutting the left third and fourth ribs and intercostal muscles with a cautery pen. The pericardium was removed, and a syringe fitted with a 30-G needle was inserted near the apex of the heart and tunneled intramuscularly to the anterior LV at the mid-ventricular level. Then, 50 μl (1.0 × 10^10^ PFU/ml) of adenovirus-expressing miR-nonsense control (miR-NC), anti-miR-153, combination of siRNA-nonsense control (siRNA-NC) and anti-miR-153 (anti-miR-153 + siRNA-NC), or combination of siRNA-Nrf2 and anti-miR-153 (anti-miR-153 + siRNA-Nrf2) were injected into from the apex of the left ventricle into the aortic root. The chest was closed in layers and a total of 0.1 ml 0.5% bupivacaine was injected subcutaneously near both edges of the skin incision to alleviate postoperative pain.

### Dual-luciferase reporter assay

The Nrf2 gene 3′-untranslated region (UTR) region containing the putative binding site of miR-153 was amplified by Polymerase chain reaction (PCR) using Pfu DNA polymerase and was cloned into the pGL3-basic reporter. Mutations in the putative binding site of miR-153 were made using Quikchange site-directed mutagenesis kit. For the reporter assays, HEK 293 cells were co-transfected with pRT-TK Renilla plasmid and pGL3-basic-Nrf2-WT or pGL3-basic-Nrf2-Mut firefly luciferase reporter plasmids as well as miR-153 mimic. Luciferase activity was measured with the Dual-Luciferase Reporter Assay System (Promega, Madison, WI) using the TD-20/20 Luminometer (Turner Designs, CA, USA).

### Animal model construction of myocardial ischemia/reperfusion injury

Adult rats were anesthetized by means of an intraperitoneal injection of sodium pentobarbital before endotracheal intubation. Heparin was administered to prevent intracoronary clot formation. As illustrated in Additional file 1: Figure S2A, B, the thorax was opened via a left lateral thoracotomy, and the pericardium removed. Coronary ligation was performed on the left coronary artery using a monofilament, placed in a polyethylene tube to create a reversible snare. After 30 min of ischemia induction, the snare was released, and the rat was reperfused for 120 min [49]. Finally, the chests were stitched, and the endotracheal tubes were removed so that the mice could resume spontaneous respiration. In addition, the sham-operated group of mice experienced the same experimental procedure except for the snare of the left anterior descending coronary arteries.

### Aspartate aminotransferase (AST), creatine kinase-MB (CK-MB) and lactate dehydrogenase (LDH), and B-type natriuretic peptide (BNP) ELISA assay

The rat serum samples from different treatment groups were collected. The serum levels of LDH, CK-MB, AST, and BNP was detected by an LDH, CK-MB, AST, and BNP ELISA kit (Roche Applied Science, Penzberg, Upper Bavaria, Germany) according to the manufacturer’s protocols, respectively. The absorbance was detected by a microplate reader (Bio-Tek Instruments, VT, USA).

### Color Doppler echocardiography

Cardiac blood flow parameters including left ventricular end-diastolic dimension (LVEDD), left ventricular end-systolic diameter (LVESD), left ventricular ejection fraction (LVEF), left ventricular fraction shortening (LVFS), left ventricular systolic pressure (LVSP), left ventricular end-diastolic pressure (LVEDP), and ± dp/dt max were detected by ultrasound Doppler examination.

### Statistical analysis

Values were presented as mean standard deviation (SD). One-way ANOVA analysis or Student’s *t* test was used for statistical analysis. *P* values < 0.05 were considered to be statistically significant.

## Supplementary information

**Additional file 1.** Additional figures.

## Data Availability

All data generated or analyzed during this study could be obtained upon reasonable request to the corresponding author.

## Abbreviations

OGD/R: Oxygen–glucose deprivation and reoxygenation
I/R: Ischemia/reperfusion
Nrf2: Erythroid 2-like 2
Bax: Bcl-2-associated X
Bcl-2: B-cell lymphoma 2
HO-1: Heme oxygenase-1
PDCD4: Programmed cell death 4
AST: Aspartate aminotransferase
CK-MB: Creatine kinase-MB
LDH: Lactate dehydrogenase
BNP: B-type natriuretic peptide
PFU: Plaque forming units
UTR: Untranslated region
GAPDH: Glyceraldehyde 3-phosphate dehydrogenase
qRT-PCR: Quantitative real-time PCR
PCR: Polymerase chain reaction
LDH: Lactate dehydrogenase
BNP: B-type natriuretic peptide
LVEDD: Left ventricular end-diastolic dimension
LVESD: Left ventricular end-systolic diameter
LVEF: Left ventricular ejection fraction
LVFS: Left ventricular fraction shortening
LVSP: Left ventricular systolic pressure
LVEDP: Left ventricular end-diastolic pressure
SD: Standard deviation
