# Correction to: Inhibition of miR‑153 ameliorates ischemia/reperfusion‑induced cardiomyocytes apoptosis by regulating Nrf2/HO‑1 signaling in rats

**DOI:** 10.1186/s12938-020-00781-4

**Published:** 2020-06-09

**Authors:** Wei Hou, Xianting Zhu, Juan Liu, Jiaguo Ma

**Affiliations:** 1 Department of Emergency, Yidu Central Hospital of Wei Fang, No.4138, South Linglongshan Road, Weifang, 262500 Shandong China; 2 Department of Nursing, Yidu Central Hospital of Wei Fang, No.4138, South Linglongshan Road, Weifang, 262500 Shandong China; 3 Department of Pediatrics, Ward 1, Yidu Central Hospital of Wei Fang, No. 4138, South Linglongshan Road, Weifang, 262500 Shandong China; 4 Department of Cardiology, Qing Zhou Traditional Chinese Hospital, No. 2727, Haidai Middle Road, Weifang, 262500 Shandong China

## Correction to: BioMed Eng OnLine (2020) 19:15 10.1186/s12938-020-0759-6

It was highlighted that the original article contained a typesetting mistake to the name of Jianguo Ma. This was incorrectly captured as Jianguo Map. This correction article shows the correct spelling of the author’s name [1].
