# Supplementary material for: Inhibition of miR-153 ameliorates ischemia/reperfusion-induced cardiomyocytes apoptosis by regulating Nrf2/HO-1 signaling in rats
Source: Biomed Eng Online. 2020 Mar 6;19:15. doi: 10.1186/s12938-020-0759-6 (PMC7059292; doi:10.1186/s12938-020-0759-6)
Supplement: Supplementary file 1 — Additional file 1. Additional figures. [file 12938_2020_759_MOESM1_ESM.docx]

**Additional Materials**


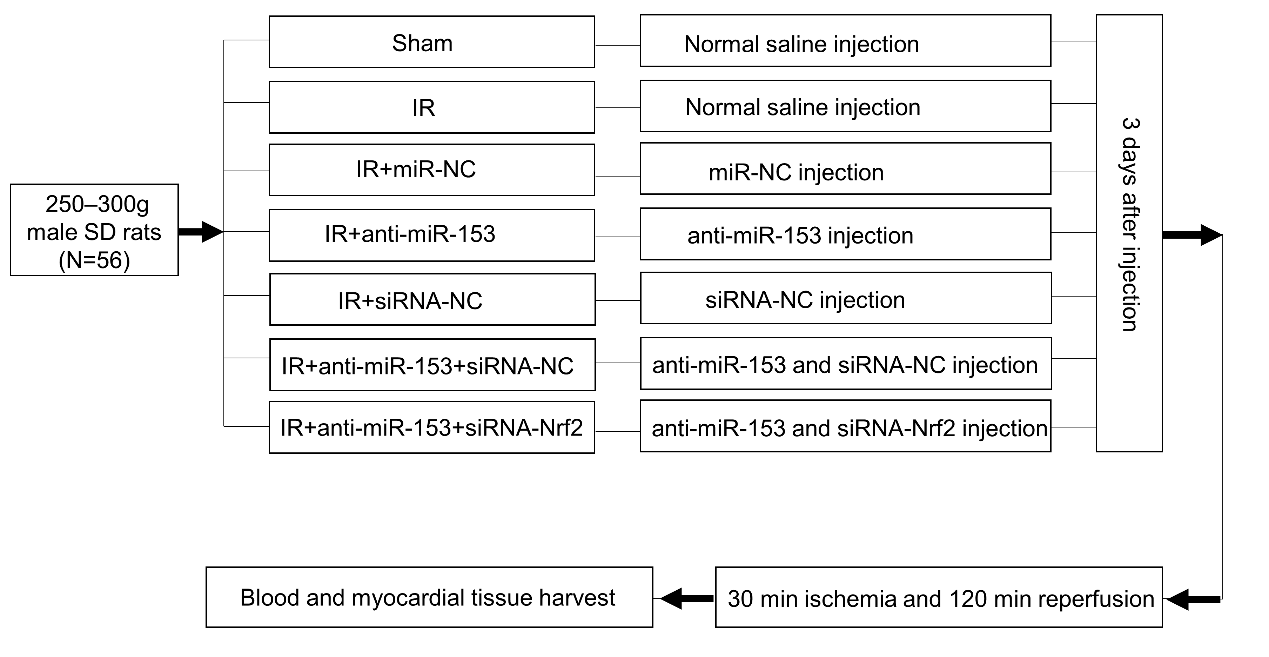


Figure S1. Experiment schematic diagram.


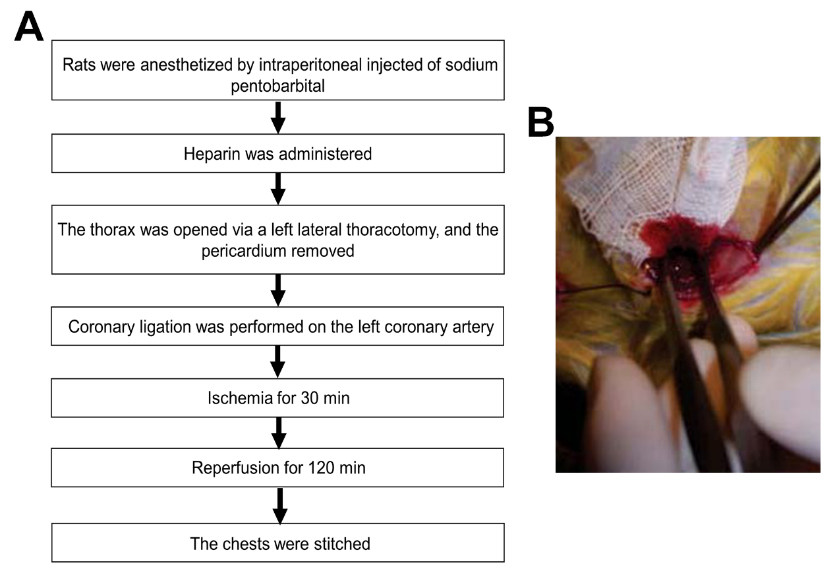


Figure S2. Schematic image which shows the procedures of the operation (A) and a photograph of the opening the left pleural cavity (B).
